# Diagnostic value of combined heart and lung ultrasound in emergency department patients with dyspnea

**DOI:** 10.1111/cpf.70009

**Published:** 2025-04-17

**Authors:** Anna Bjällmark, Gustaf Hummel, Kambiz Shahgaldi

**Affiliations:** ^1^ Department of Clinical Diagnostics, School of Health and Welfare Jönköping University Jönköping Sweden; ^2^ Department of Cardiology Danderyd Hospital Danderyd Sweden; ^3^ Department of Clinical Physiology Danderyd Hospital Danderyd Sweden; ^4^ Karolinska Institutet, Department of Clinical Sciences Danderyd Hospital Danderyd Sweden

**Keywords:** diagnostic accuracy, heart failure, pneumonia, point‐of‐care ultrasound, rapid diagnosis, ultrasonography

## Abstract

**Background:**

Acute dyspnea in emergency departments (ED) requires prompt and accurate diagnosis due to its high mortality and readmission rates. Conventional diagnostic methods are resource‐intensive and time‐consuming. This study aimed to evaluate the diagnostic accuracy and time to diagnosis of combined heart and lung ultrasound (HeaLus) compared to standard emergency department evaluation in patients presenting with dyspnea.

**Methods:**

A prospective study was conducted in a cohort of 61 patients at the ED of Danderyd Hospital, Sweden. HeaLus examinations were performed alongside routine investigations. Diagnostic performance of HeaLus and ED evaluation was assessed for accuracy, sensitivity, specificity, positive predictive value, and negative predictive value, and agreement using Kappa index. Median time to diagnostics was compared between HeaLus and ED evaluation using Mann‐Whitney U‐test.

**Results:**

Heart failure was the most common diagnosis (20%) among patients presenting with dyspnea. The diagnostic accuracy, sensitivity, specificity, positive predictive value, and negative predictive value were 95% (95% CI: [87%, 98%]), 98% (95% CI: [88%, 100%]), 90% (95% CI: [69%, 97%]), 95% (95% CI: [85%, 99%]), and 94% (95% CI: [74%, 99%]), respectively. The agreement between HeaLus and ED diagnoses was 0.88. Time to diagnosis was significantly reduced with HeaLus (21 min vs. 3 h and 28 min).

**Conclusions:**

HeaLus offers rapid and accurate assessment of dyspnea. These results suggest that HeaLus could be valuable in optimizing patient management, particularly in settings with limited resources and long ED wait times.

## 1 INTRODUCTION

Acute dyspnea is a widespread manifestation encountered in emergency departments (ED) and can be attributed to a multitude of underlying factors and conditions (Parshall et al., 2012). Prompt and accurate diagnosis is a crucial factor, as previous research has shown that this patient group has high mortality and readmission rates (Sørensen et al., 2021). Currently, these patients undergo conventional diagnostic assessments, including history and physical examination, standard laboratory tests, electrocardiography and/or radiographic imaging to identify the etiology of dyspnea (Martindale et al., 2016). This approach can be both resource‐intensive and time‐consuming and may lead to delayed initiation of appropriate treatment.

Echocardiography is a well‐established diagnostic technique in clinical practice. Meanwhile, lung ultrasound has shown to be a valuable diagnostic tool, reducing the time to diagnosis (D. A. Lichtenstein & Mezière, 2008). The combined utilization of heart and lung ultrasound (HeaLus) offers enhanced clinical value in the determination of the underlying cause of dyspnea, as it enables differentiation between cardiac and pulmonary origins of the condition. There is a growing body of evidence supporting the usefulness of combined HeaLus in the evaluation of patients with cardiorespiratory symptoms, particularly in diagnosing patients with acute heart failure (Dehbozorgi et al., 2019; Farahmand et al., 2020; Öhman et al., 2019; Russell & Ehrman, 2017; Russell et al., 2015) or to establish the etiology of dyspnea (Gaber et al., 2019; Gallard et al., 2015; Papanagnou et al., 2017; Zanobetti et al., 2017). A systematic review and meta‐analysis recently conducted demonstrated that the utilization of point‐of‐care ultrasound in patients with acute dyspnea results in improved diagnostic accuracy and outcomes in comparison to conventional diagnostic approaches (Szabó et al., 2022). It has been suggested that patients admitted with dyspnea should be managed by ultrasound in the ED as a standard to improve patient care. Given these promising findings, it is essential to further investigate the impact of HeaLus to ensure its effectiveness and reliability before incorporating it into standard clinical guidelines. Therefore, the aim of this study was to evaluate the diagnostic performance and the time to diagnosis of combined HeaLus as compared to ED evaluation in patients in the ED with dyspnea.

## 2 METHODS

### 2.1 Study design

This was a prospective HeaLus study in ED patients with undifferentiated dyspnea. The study was conducted according to the declaration of Helsinki and was approved by the Swedish Ethical Review Authority (2020‐00182). All participants gave written informed consent to participate.

### 2.2 Study population

During December 2020 to March 2021, nonconsecutive patients were recruited from the ED of Danderyd Hospital in Sweden. Patients were enrolled based on the availability of a trained physician in HeaLus. Inclusion criteria were age ≥18 years with a primary complaint of dyspnea. Exclusion criteria (added during the study) was patients with known Covid‐19 with acute respiratory deterioration. This was to avoid exposing the examiner to unnecessary infection risk in nonurgent situations.

### 2.3 Study procedure

The HeaLus examinations were performed either while the patient was waiting for routine investigations or during the waiting period for the results of such investigations. The routine investigations included vital sign assessment, medical history, physical examination, electrocardiography, chest X‐ray, computed tomography scan, and/or blood samples such as NT‐pro‐BNP when needed. Before the examination started, the physician reviewed the ED documentation about the patient's vital signs. The examination was performed on the existing hospital bed in the ED with the head end elevated at an angle of approximately 30‐45 degrees or in a sitting position, as some patients needed this based on their general condition and respiratory status. No active patient history was taken by the physician. The examinations were saved in the hospital Picture Archiving and Communication System (PACS). One experienced physician was involved in the acquisition of ultrasound images and the interpretation of the associated results. The physician was blinded to the results from standard ED evaluation. The duration for ultrasound examination as well as for analyzing the ultrasound images was also assessed. This time did not include the triage process.

One physician determined the final diagnosis for undifferentiated dyspnea by carefully reviewing the medical records of each patient. This review was conducted without knowledge of the ultrasound examination results. Information on the time of arrival at the emergency department, the time to decisive examination results (laboratory and radiology), and the time to decisive notes were also noted. This allowed for tracking of the time to diagnosis.

### 2.4 Cardiac ultrasound

Cardiac ultrasound was performed using a GE Vivid S70 ultrasound machine with a phased array transducer (M5Sc‐D) utilizing a frequency range of 1.2–3.7 MHz (GE, Oslo, Norway). The echocardiographic examination was performed according to the current guidelines (Baumgartner et al., 2017; Lang et al., 2015; Nagueh et al., 2016; Rudski et al., 2010; Zoghbi et al., 2017) and consisted of subcostal, parasternal long‐ and short‐axis views, and apical four‐, three‐, and two‐chamber views. Morphological assessment of the heart valves was performed to detect or exclude findings consistent with possible significant stenosis, insufficiency, and potential prolapse. Simple screening with color Doppler over the heart valves was carried out to detect or exclude major valve insufficiencies. The E/A ratio over the mitral inflow was used for assessment of diastolic function and filling pressure. Tissue Doppler velocities were measured in the septal and lateral walls for the assessment of diastolic function. The estimated right ventricular pressure was obtained through tricuspid regurgitation. Acceleration time over the pulmonary valve was estimated, and longitudinal right ventricular function was assessed (TAPSE). The presence or absence of right ventricular involvement was noted, and if present, an assessment of pressure or volume overload was made. The central venous pressure was estimated via the inferior vena cava (IVC) and its collapse. The presence or absence of pericardial effusion was noted, and if present, an assessment of hemodynamic impact was performed. The systolic left ventricular function was estimated visually, as hyperdynamic, normal, impaired, moderately impaired, or severely impaired.

### 2.5 Lung ultrasound

Lung ultrasound was performed using a GE Vivid S70 ultrasound machine (GE, Oslo, Norway) with linear transducer (9L‐D), or when needed, with a phased array transducer (M5Sc‐D), together with dedicated software. The frequency range of the linear transducer was 8.4–9 MHz and 1.2–3.7 MHz for the phased array transducer.

**Figure 1:**
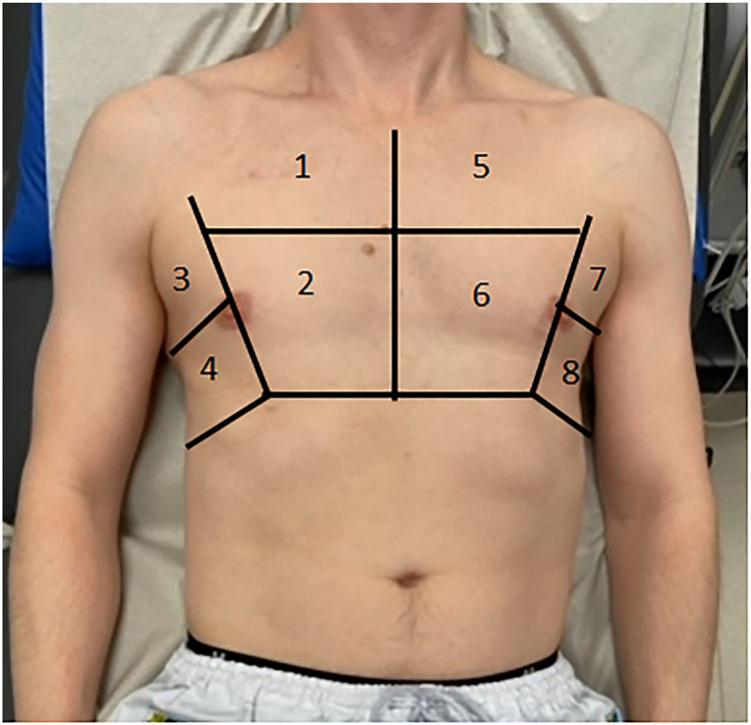
Illustration of the 8‐zone lung ultrasonography.

The examination protocol was performed according to current recommendation (Volpicelli et al., 2012) and consisted of a lung ultrasound comprising of four examination points/zones on each hemithorax, totaling eight examination points (Figure 1). An upper anterior and lower anterior/lateral point and an upper lateral and lower lateral/posterior point bilaterally were chosen to avoid the heart in accordance with the BLUE‐protocol (D. A. Lichtenstein, 2015; D. A. Lichtenstein & Mezière, 2008). The upper anterior point (z1 (right side) and 5 (left side) is located over an area where any gas in a previously healthy lung patient is likely to be found in case of a pneumothorax, that is, at the top. The lower anterior/lateral point (z2 and 6) corresponds to an area where lung mobility is highest, thereby optimizing the likelihood of visualizing the movement of the pleural surfaces. The lower lateral/posterior point (z4 and 8) corresponds to the PLAPS view (posterolateral alveolar and/or pleural syndrome) (D. A. Lichtenstein, 2015; D. A. Lichtenstein & Mezière, 2008), where the highest percentage of consolidations is found (Nazerian et al., 2015). An upper lateral point (z3 and 7) was also included in the examination protocol to better estimate any fluid leakage, that is, for a simple estimation of the amount of a potential pleural effusion and for quantifying the number of B‐lines over multiple areas (Ibitoye et al., 2018; Picano & Pellikka, 2016; Volpicelli et al., 2012). The transducer was placed in an intercostal space and held as perpendicularly as possible against the chest wall. One scan was performed per point/zone. The assessment conducted was the presence/absence of pleural sliding. If there was an absence of pleural sliding, the presence of a lung point was searched for. The presence/absence of A‐lines, B‐lines, and, in case of the presence of B‐lines, an evaluation of their number, location, and movement pattern was performed. The presence/absence of fluid in the pleural space was also assessed, with a simple estimation of the amount, as well as an evaluation of the homogeneity/heterogeneity of the fluid content. The presence of consolidation was noted, and in such cases, a closer evaluation was performed to determine whether the findings were consistent with atelectasis or pneumonia, through the presence of static or dynamic air bronchograms (D. Lichtenstein et al., 2009). The presence of rounded or triangular pleural lesions as a sign of pleural infection/consolidation was also noted.

### 2.6 Statistical analysis

Data are presented as mean ± standard deviation (SD) or median (interquartile range (IQR)). Diagnostic accuracy, sensitivity, specificity, positive predictive value, and negative predictive value were calculated with Wilson score 95% confidence interval (CI). Difference in median time to diagnostics between HeaLus and of ED evaluation was tested using Mann‐Whitney U‐test. Agreement between findings obtained from HeaLus and the final ED diagnosis was performed using the Kappa index. Statistical analysis was performed using Microsoft Excel (IBM, United States), and Stata 18.0 (StataCorp, United States).

## 3 RESULTS

During a period of 3 months, a total of 61 (40 males and 21 females) patients admitting to ED with dyspnea as their symptom were enrolled in this study. The mean age of the patients was 64 ± 19 years, with the youngest being 20 years old and the oldest being 91 years old. No patients were excluded during the analysis phase. Four patients had to discontinue the study due to serious finding that needed immediately care (e.g., a young patient with pulmonary embolism with severe right ventricular impairment). Table 1 presents the characteristics of the patients included in the study.

**Table 1:** Patient characteristics.

Characteristics	Value
Age (yr), mean ± SD	64 ± 19
Female, *n* (%)	21 (34)
Heart rate, beats/min ± SD	79 ± 16
Systolic blood pressure, mmHg ± SD	135 ± 26
Diastolic blood pressure, mmHg ± SD	82 ± 17
Heart failure, *n* (%)	12 (20)
Chronic obstructive lung disease, *n* (%)	11 (18)
Hypertension, *n* (%)	25 (41)
Diabetes mellitus, *n* (%)	7 (11)

The most common cause of dyspnea was heart failure (20%), with global LV systolic function visually assessed and presence of B‐lines, while pneumonia/Covid‐19 (10%) with presence of lung sliding and B‐lines (unilateral and/or bilateral) was the most common etiology on the lung side. A description of the etiologies is provided in Table 2. In total, thirty patients showed signs of lung abnormalities, three patients had absence of lung sliding, twenty‐five patients showed signs of elevated B‐lines, nineteen patients had pleura effusion and seven patients had lung consolidation.

**Table 2:** The most common etiologies, observed in 46 of the 61 patients included in the study. Note that no patient had a combination of different diagnoses.

Diagnosis	*N* (%)
Heart failure	12 (20)
Dyspnea unspecified	7 (11)
Pneumonia caused by Covid‐19	6 (10)
Pneumonia	5 (8)
Chronic obstructive pulmonary disease exacerbation	5 (8)
Pulmonary embolism	5 (8)
Pulmonary embolism with right ventricular involvement	3 (5)
Pneumothorax	3 (5)

The agreement (Kappa index) between the HeaLus and ED diagnoses was 0.88, indicating a very good agreement. The diagnostic accuracy, sensitivity, specificity, positive predictive value, and negative predictive value were 95% (95% CI: [87%, 98%]), 98% (95% CI: [88%, 100%]), 90% (95% CI: [69%, 97%]), 95% (95% CI: [85%, 99%]), and 94% (95% CI: [74%, 99%]), respectively.

The median examination time for combined HeaLus was 18 min (IQR: 17–22), with a median analysis time of 3 min (IQR: 1–4). The median time to diagnosis for combined HeaLus was 21 min (IQR: 19–25), while the median time to ED diagnosis was 3 h and 28 min (IQR: 55 min to 5 h and 58 min) (*p* < 0.001). During the ED stay the study cohort underwent a total of 29 computed tomography scans and six conventional radiographs.

## 4 DISCUSSION

Managing patients with dyspnea in emergency departments is a challenging task, given the nonspecific nature of the symptoms. Patients experiencing dyspnea due to heart failure often exhibit symptoms similar to those with dyspnea caused by lung disease. This underscores the importance of accurately identifying the etiology of dyspnea to enable correct treatment. Our study shows that HeaLus can be a valuable tool for managing patients with dyspnea in the ED department by demonstrating relatively high values on diagnostic accuracy (95%), sensitivity (98%), specificity (90%), positive predictive value (95%), and negative predictive value (95%). These findings are consistent with previously reported values utilizing the combination of IVC, cardiac and lung ultrasound for managing patients in the ED (Bataille et al., 2014; Carlino et al., 2018; Dehbozorgi et al., 2019; Farahmand et al., 2020; Gaber et al., 2019; Kajimoto et al., 2012; Öhman et al., 2019; Russell et al., 2015; Sforza et al., 2017; Silva et al., 2013; Zanobetti et al., 2017). When considering the collective outcomes of these studies, the median values (range) for accuracy, specificity, and sensitivity were 84% (38–96%), 97% (76–100%), and 83% (32–100%), respectively. Median values (range) for positive predictive value and negative predictive value were 92% (29–100%) and 94% (61–100%). Variations in the results between the studies may be due to different comparative modalities used in the various studies. For instance, one study utilized plasma brain natriuretic peptide (BNP) levels as the diagnostic criterion for patients with acute decompensated heart failure (Farahmand et al., 2020). In contrast, another study, focusing on the same patient cohort, incorporated a broader range of data, encompassing laboratory results, radiographs, echocardiograms, admission notes, and discharge summaries to establish the final diagnosis (Dehbozorgi et al., 2019). The accuracy of the diagnosis may also be influenced by the underlying cause of the patient's dyspnea, as previous studies have shown varying diagnostic accuracies observed for different conditions (Bataille et al., 2014; Gaber et al., 2019).

Rapid diagnosis is crucial for patients with dyspnea, considering the associated high mortality and readmission rates (Sørensen et al., 2021). This study showed that the median time to diagnosis with HeaLus was remarkably shorter (21 min (IQR: 19–25)) compared to standard ED evaluation (3 h and 28 min (IQR: 55 min to 5 h and 58 min)), suggesting that HeaLus could accelerate decision‐making and improve patient outcomes. Several other studies reported that combined ultrasound led to faster diagnostics (Dehbozorgi et al., 2019; Gaber et al., 2019; Gallard et al., 2015; Kajimoto et al., 2012; Russell et al., 2015; Zanobetti et al., 2017), however, only few of these studies included time data for the standard examination pathway, with durations of 244 min (128–360 min) and 186 ± 72 min (Gaber et al., 2019; Zanobetti et al., 2017). The use of ultrasound is not only faster but also reducing radiation exposure and is noninvasive, making it a safe option for patients.

Many hospitals face challenges related to a shortage of available hospital beds and prolonged wait times at emergency departments. The standardized emergency care process involves multiple examinations and tests, leading to delays in receiving test results. When healthcare resources cannot meet societal needs, it is crucial to employ time‐saving, patient‐safe, and cost‐effective methods. Despite echocardiography being established, lung ultrasound, recognized for its speed and simplicity, is underutilized, and rarely used by emergency physicians (Wimalasena et al., 2018). Lung ultrasound has proven advantageous for less experienced healthcare professionals. It has been demonstrated that clinicians, after undergoing a short training session, can acquire and interpret lung ultrasound images with high quality (Chiem et al., 2015). By incorporating lung ultrasound, significant pathologies can be detected, allowing for timely and appropriate diagnosis and treatment. This study provides evidence supporting the potential integration of HeaLus into clinical practice, especially in emergency settings. The potential economic benefits and efficiency gains associated with HeaLus may contribute to reducing healthcare costs and optimizing resource utilization.

## 5 LIMITATIONS

The study is constrained by a small cohort of patients admitted to the ED with dyspnea, as well as the limited number of patients within each disease group. Another limitation relates to the availability of the physician who conducted the HeaLus, which affected the enrollment process. This may have introduced a selection bias, potentially impacting the generalizability of the results. Additionally, the exclusion criteria introduced during the study, which excluded patients with known Covid‐19 with acute respiratory deterioration, imposed certain restrictions on the eligible patient population.

Moreover, the lack of multiple evaluators for determine ED diagnosis could impact the reliability and robustness of the findings. However, it is important to note that the physician determining the final diagnosis was blinded to the ultrasound results, which helps mitigate the risk of diagnostic bias. The reference gold standard in our study, which was the standard ED procedure for dyspnea may not be robust enough, as it relies on clinical, laboratory, and radiology assessments that can vary. However, it reflects the real‐world diagnostic process in emergency care, where decisions are made based on a combination of these modalities.

It is important to recognize that all ultrasound technologies are operator‐dependent, and their effectiveness is well established when performed by trained individuals. However, it remains to be determined whether this technology can be broadly applied. While the study provides useful insights, it has several limitations, and its findings should be validated through larger, more controlled studies to ensure the applicability of HeaLus in the clinical setting.

## 6 CONCLUSION

The combined utilization of HeaLus shows potential as a valuable diagnostic tool that may enhance the clinical assessment of the underlying cause of dyspnea and may contribute to a more efficient diagnosis and initiation of appropriate treatment. Its use in the ED can improve patient care and should be considered as a standard diagnostic tool for patients presenting with dyspnea. Further studies are needed to validate these findings and to establish guidelines for the use of ultrasound in the evaluation of patients with acute dyspnea.

## CONFLICT OF INTEREST STATEMENT

The authors declare no conflicts of interest.

## Data Availability

The data in this study is not publicly available. However, access to the data will be granted upon reasonable request to the corresponding author.
